# Impact on prognosis of rebiopsy in advanced non-small cell lung cancer patients after epidermal growth factor receptor-tyrosine kinase inhibitor treatment: a systematic review

**DOI:** 10.1186/s12885-019-5309-x

**Published:** 2019-01-25

**Authors:** Takuma Imakita, Hirotaka Matsumoto, Katsuya Hirano, Toshiyuki Morisawa, Azusa Sakurai, Yuki Kataoka

**Affiliations:** 1 Department of Respiratory Medicine, Hyogo Prefectural Amagasaki General Medical Center, Amagasaki, Hyogo Japan; 2 Department of Gastroenterology, Hyogo Prefectural Amagasaki General Medical Center, Amagasaki, Hyogo Japan

**Keywords:** Non-small cell lung cancer, Epidermal growth factor receptor-tyrosine kinase inhibitor, T790M mutation, Rebiopsy

## Abstract

**Background:**

Osimertinib, the third-generation epidermal growth factor receptor-tyrosine kinase inhibitor (EGFR-TKI), has become the standard treatment in cases where rebiopsy reveals T790M mutation after the first-line EGFR-TKI treatment. However, the prognosis of patients after rebiopsy, the most important outcome for cancer patients, has not been described sufficiently. This systematic review aimed to clarify whether rebiopsy contributes to improved prognosis in the first- or second-generation EGFR-TKI refractory patients.

**Methods:**

Using free word and control terms related to “non-small cell lung cancer” and “rebiopsy,” we searched studies from Medical Literature Analysis and Retrieval System Online via PubMed, Embase, Cochrane Central Register of Controlled Trials, and World Health Organization International Clinical Trials Registry Platform. We included cohort studies and case reports written in English and judged whether each study answers our research questions.

**Results:**

Of the 144 studies included, only one reported the prognosis of patients with/without rebiopsy showing that in EGFR-TKI refractory non-small cell lung cancer patients, the post-progression survival (PPS) was significantly longer in patients who received rebiopsy and treatment based on a resistant mechanism (median PPS 24.2 months) than those who received rebiopsy and salvage regimen (median PPS 15.2 months, *p* = 0.002) and who did not receive rebiopsy (median PPS 9.7 months, *p* < 0.001). Most of the other studies reported the detection rate of T790M mutation or rebiopsy procedure.

**Conclusions:**

Only a few previous studies have investigated the effectiveness of rebiopsy. Hence, further study is needed to determine the prognosis or adverse events of rebiopsy.

## Background

A standard care for non-small cell lung cancer (NSCLC) with epidermal growth factor receptor (EGFR) gene mutation is administration of EGFR-tyrosine kinase inhibitors (TKIs). The progression free survival (PFS) of patients treated with EGFR-TKIs is significantly longer than that of those who received chemotherapy [1–3]. T790M mutation is known to be the most common acquired resistance mechanism to first- or second-generation EGFR-TKIs [4, 5]. Osimertinib is a third-generation EGFR-TKI. It has become the standard treatment in cases where rebiopsy reveals T790M mutation after first-line treatment with EGFR-TKIs [6, 7]. After the tumors develop resistance to EGFR-TKIs, rebiopsy of the tissue or liquid biopsy (plasma or urine sampling) [8] to identify T790M mutation plays an important role in deciding the next line of treatment [6, 7].

Although the duration of survival is one of the most important outcomes for lung cancer patients [9], only a few studies on rebiopsy have evaluated it. A previous systematic review by Luo et al. [10] reported the diagnostic accuracy of a particular method of EGFR mutation detection, but patients’ survival rates were not described.

This study aimed to clarify if performing rebiopsy contributes to improve prognosis in the first- or second-generation EGFR-TKI refractory patients through a systematic review of previous literature.

## Methods

### Protocol and registration

We followed the Preferred Reporting Items for Systematic Reviews and Meta-Analyses (PRISMA) statement during all stages of design, implementation, and reporting [11]. Our protocol was registered in the International Prospective Register of Systematic Reviews (ID: CRD42017068630). PRISMA checklist is shown in supplement 1.

### Eligibility criteria

We included cohort studies and case reports written in English and excluded review articles. We included articles irrespective of publication status.

### Information sources

Using free word and control terms related to “non-small cell lung cancer” and “rebiopsy,” we searched studies from Medical Literature Analysis and Retrieval System Online via PubMed, Embase, Cochrane Central Register of Controlled Trials, and World Health Organization International Clinical Trials Registry Platform.

### Search

The search queries are shown in supplement 2.

### Analytic framework

**Fig. 1:**
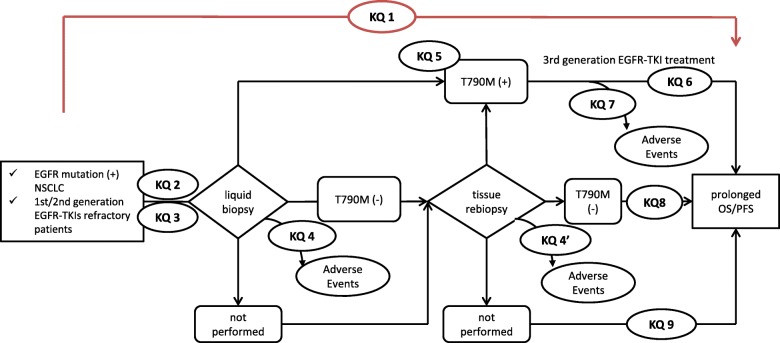
PRISMA flow diagram

We developed an analytic framework (AF) according to the recommendation of the Agency for Healthcare Research and Quality [12]. Panel members, including an oncologist (TM) and two pulmonologists (YK and KH), with over 10 years of experience were involved in the discussion and in reaching a consensus. The AF contains key questions (KQs). KQ1: Does rebiopsy in the first- or second-generation EGFR-TKI refractory patients improve prognosis? KQ2: What are the rebiopsy samples? KQ3: How are EGFR gene mutations detected? KQ4: Is the benefit of rebiopsy greater than the potential harm? KQ5: How often is T790M mutation detected in the first- or second-generation EGFR-TKI refractory cases? KQ6: How is the prognosis when the third-generation EGFR-TKI is administered to T790M-positive cases? KQ7: Is the benefit of the third-generation EGFR-TKI to T790M-positive cases greater than the potential harm? KQ8: How is the prognosis of T790M-negative cases? KQ9: How is the prognosis when rebiopsy is not performed in the first- or second-generation EGFR-TKI refractory cases? (Fig. 1).

### Study selection

Two researchers (TI and TM) independently screened the titles and abstracts of articles identified during the initial search, and assessed the eligibility based on full-text reviews. Disagreements were resolved by discussion between the researchers. If necessary, another researcher (YK) acted as an arbiter.

### Data collection process

We created a data extraction sheet. For each included review, one researcher (TI) extracted the data and judged whether the study answered the key questions. Other researchers (YK and AS) confirmed these. Disagreements were resolved by discussion between the researchers.

### Data items

We extracted the titles, authors, journal, published year, abstract, and publication style from each article.

### Risk of bias in individual studies

We did not assess the risk of bias in individual studies.

### Summary measures

The primary outcome was the number of studies that answer each key question.

### Synthesis of results

We summarized the results using descriptive statistics.

## Results

**Fig. 2:**
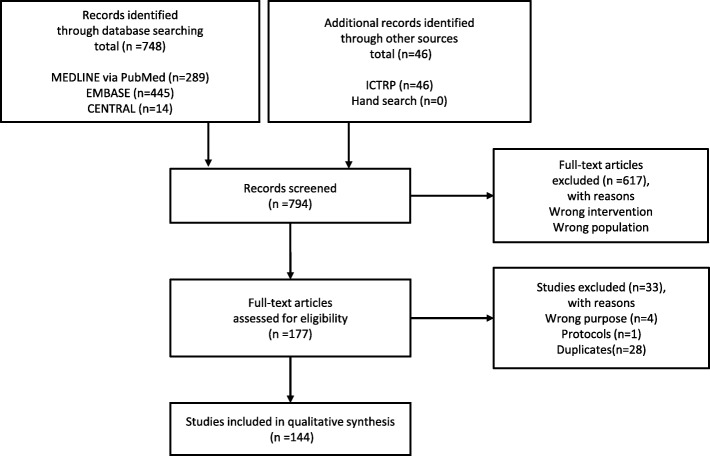
Analytic framework (EGFR, epidermal growth factor; TKI, tyrosine kinase inhibitor; NSCLC, non-small cell lung cancer; OS, overall survival; PFS, progression free survival)

A total of 794 articles were screened. After assessing the eligibility based on a full-text review, we included 144 articles in qualitative synthesis (Fig. 2). The included articles are listed in supplement 3. The number of studies which answer each KQ are shown in Table 1. The rebiopsy samples were reported in 98 (68%) studies. The most common samples were: lung lesions, lymph nodes, plasma, pleural fluid, and other metastatic lesions. The methods used for detecting EGFR mutations were reported in 93 (65%) studies. Droplet digital polymerase chain reaction, amplification refractory mutation system, and next-generation sequencing were the most common detection methods. The detection rate of T790M mutation was reported in 99 (69%) studies. However, only a few studies reported on the prognosis of patients (*n* = 1, 0.7%), adverse events of rebiopsy (*n* = 9, 6.3%), and those with third-generation EGFR-TKI (*n* = 1, 0.7%). Only Zhang et al.’s study reported the prognosis of patients [13]. They reviewed 227 EGFR-TKI refractory NSCLC patients and reported that the post-progression survival (PPS) was significantly longer in patients who received rebiopsy and treatment based on a resistant mechanism (*n* = 70, median PPS 24.2 months) than those who received rebiopsy and salvage regimen (*n* = 37, median PPS 15.2 months, *p* = 0.002) and who did not receive rebiopsy (*n* = 120, median PPS 9.7 months, *p* < 0.001).

**Table 1:** The primary outcome

		n
KQ 1	Does rebiopsy in the 1st or 2nd generation EGFR-TKI refractory patients improve prognosis?	1
KQ 2	What are the rebiopsy samples?	98
KQ 3	How are EGFR gene mutations detected?	93
KQ 4	Is the benefit of liquid biopsy greater than the harm?	1
KQ 4’	Is the benefit of tissue rebiopsy greater than the harm?	8
KQ 5	How often is T790M mutation detected in the 1st or 2nd generation EGFR-TKI refractory cases?	99
KQ 6	How is the prognosis when the 3rd generation EGFR- TKI is administered to T790M positive cases?	11
KQ 7	Is the benefit of the 3rd generation EGFR-TKI to T790M positive cases greater than the harm?	1
KQ 8	How is the prognosis of T790M negative cases?	29
KQ 9	How is the prognosis when rebiopsy is not performed in the 1st or 2nd generation EGFR-TKI refractory cases?	1

The key questions (KQs) and the number of articles which answer to each KQ are shown

## Discussion

In this systematic review, we developed an analytic framework to assess whether performing rebiopsy improves the prognosis in the first- or second-generation EGFR-TKI refractory patients and found several appropriate studies. The results showed that methods (e.g., biopsy samples, technique, and test kit) and success rate of rebiopsy and positive detection rate of T790M mutation were well described in previous studies. However, only a few studies were conducted to clarify the effectiveness of performing rebiopsy; only one investigated whether performing rebiopsy improves the prognosis (KQ1). Most of the included studies were diagnostic test accuracy (DTA) studies.

In order to argue the effectiveness of rebiopsy, it is important to investigate patient prognosis when rebiopsy is or is not performed. Although DTA studies reported surrogate outcomes (e.g., positive rate of T790M mutation), they sometimes have no linkage with clinical outcomes (e.g., prognosis) [14]. For example, Presley et al. reported that broad-based genomic sequencing for advanced lung cancer was not significantly associated with 12-month mortality [15]. Lim et al. revealed that there was no significant difference in overall survival and progression-free survival between a T790M mutation-positive group and a T790M mutation-negative group after the first EGFR-TKI treatment failed [16].

Only the prognostic study conducted by Zhang et al. had several critical biases. First, although the general condition of the patients is critical information for a prognostic study [17], these were not referred to in this study. The patients not receiving rebiopsy may have had a poorer prognosis because potentially, those with a poor general condition may not have been indicated for rebiopsy. The reasons for not performing rebiopsy should have been noted. Second, patients’ general condition or the next regimen after failure of first EGFR-TKI treatment can be confounding factors, which were not considered in the study. A new study, designed to investigate the survival rates when rebiopsy is/is not performed, and in which adequate follow-up time and confounding factors are taken into consideration is needed. This will clarify the prognostic benefit of performing rebiopsy and its cost-effectiveness.

The number of studies reporting the adverse events associated with the rebiopsy procedure was limited, which makes drawing conclusions regarding the benefits of rebiopsy difficult. Reporting guidelines are widely used according to the study types. After the Consolidated Standards of Reporting Trials statement [18] was published, the quality of randomized control studies in oncology showed suboptimal improvement [19]. To assess the usefulness of the intervention, we should follow the Standards for Reporting Diagnostic Accuracy statement [20] and investigate adverse events — which will lead to improvements in the quality of prognostic studies.

This study had some limitations. First, the articles published after the targeted searching period are not included in this review. The only study investigating the prognosis was published as an abstract presented at a congress more than 2 years ago. Second, the extraction of data was performed by one researcher. It may be better to reduce bias through independent extraction of data by two researchers. Third, the AF of this study lacked the viewpoint of patients. However, it is reasonable according to the statement from American Society of Clinical Oncology [21], which indicated that the key elements of framework are the clinical benefits (e.g., hazard ratio for death, overall survival, and progression-free survival) and toxicity (e.g., adverse effects).

## Conclusions

With regard to rebiopsy of EGFR-TKI refractory NSCLC, studies reporting the survival rates of patients or adverse events of the rebiopsy procedure are limited compared to DTA studies. Hence, further studies on the prognosis or adverse events associated with rebiopsy are needed to investigate the effectiveness of rebiopsy.

## Abbreviations

AF: Analytic framework
DTA: Diagnostic test accuracy
EGFR-TKI: Epidermal growth factor receptor-tyrosine kinase inhibitor
KQ: Key question
NSCLC: Non-small cell lung cancer
PFS: Progression free survival
PRISMA: Preferred Reporting Items for Systematic Reviews and Meta-Analyses
